# Molecular Recognition and Modification Strategies of Umami Dipeptides with T1R1/T1R3 Receptors

**DOI:** 10.3390/molecules30132774

**Published:** 2025-06-27

**Authors:** Kaixuan Hu, Guangzhou Sun, Wentong Yu, Mengyu Zhang, Shuang Wang, Yujie Cao, Dongling Hu, Li Liang, Gang He, Jianping Hu, Wei Liu

**Affiliations:** 1 Bamboo & Forest Institute of Science, Technology and Industrial Innovation, Leshan Normal University, Leshan 614004, China; 13187111298@163.com; 2 Key Laboratory of Medicinal and Edible Plants Resources Development of Sichuan Education Department, School of Pharmacy, Chengdu University, Chengdu 610106, China; s19196482291@163.com (G.S.); 17711398348@163.com (W.Y.); octopuszmyy@163.com (M.Z.); w2397740082@163.com (S.W.); c18942879157@163.com (Y.C.); 19848530317@163.com (D.H.);

**Keywords:** umami dipeptide, T1R1/T1R3, molecular dynamics simulation, molecular recognition, molecular modification

## Abstract

Umami is a fundamental taste sensation, often described as a delicious and pleasant flavor perception. To enhance or complement the original flavor and meet the tastes of diverse regions, umami dipeptides have been extensively utilized in global food manufacturing. Currently, the application and purification techniques of dipeptides are relatively mature, while their umami mechanisms and molecular modification are both scarce. In this work, the 3D structure of the umami dipeptide target T1R1/T1R3 was first obtained through sequence alignment and homology modeling, then followed by the successful construction of a database containing 400 samples of dipeptides. Subsequently, the complex models of T1R1/T1R3, respectively, with DG (Asp-Gly) and EK (Glu-Lys) (i.e., T1R1_DG/T1R3, T1R1/T1R3_DG, T1R1_EK/T1R3, and T1R1/T1R3_EK) were obtained via molecular docking and virtual screening. Finally, based on comparative molecular dynamics (MD) simulation trajectories, the binding free energy was calculated to investigate receptor–ligand recognition and conformational changes, providing some implications for potential modifications of umami dipeptides. T1R1 tends to bind relatively small umami dipeptides, whereas T1R3 does the opposite, both of which favor the recognition of acidic and hydrophilic dipeptides. By comparing strategies such as hydroxyl introduction and chain length alteration, electrostatic effects may be more important than non-polar effects in molecular design. This work not only explores the recognition mechanism of umami dipeptides with the receptor T1R1/T1R3 showing certain theoretical significance, but also provides feasible suggestions for dipeptide screening and modification having certain application value.

## 1. Introduction

**Figure 1:**
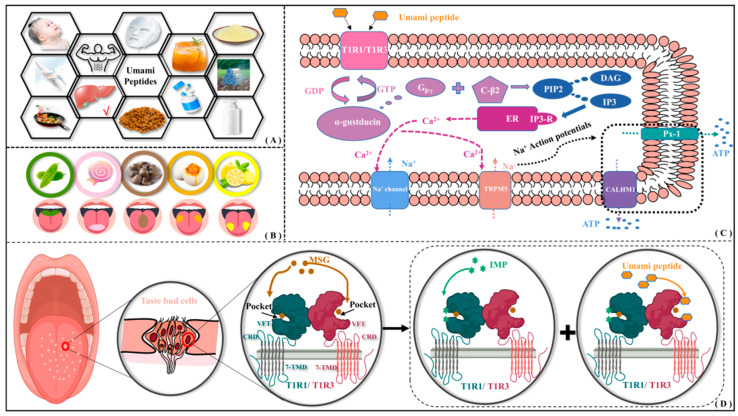
Application of umami peptides and their possible perceptual mechanism. (**A**) Wide application of umami peptides. (**B**) Perceptual distribution of five tastes on tongue surface. (**C**) Intracellular signaling pathway of umami perception. (**D**) Molecular recognition of umami dipeptides and their receptors.

As a key ingredient introducing unique taste and flavor profiles, natural umami dipeptides are found in various food products including alcohol, seafood, meat, soy sauce, mushrooms, beans, and so on. In addition to being a food additive, umami dipeptides also possess numerous health benefits, such as reducing fat deposition and plasma leptin levels [1], regulating gastrointestinal function [2], decreasing dietary sodium intake, and lowering the risk of stroke and coronary heart disease [3], etc. Therefore, umami dipeptides have also been extensively utilized in pharmaceutical, flavoring, insect repellent, beverage, and the health care and skin care industries [4] (see Figure 1A). Umami is characterized by adding richness to food texture and enhancing pleasurable qualities and is also recognized as the fifth fundamental taste sensation after sour, sweet, bitter, and salty (see Figure 1B).

In 1908, the Japanese scientist Ikeda first discovered the umami substance glutamate from dried seaweed using a crystallization method [5]. Subsequently, Kodama and Kuninaka, respectively, extracted two umami nucleotides—5′-inosinic acid (IMP) and 5′-guanylic acid (GMP)—from dried bonito and shiitake soup, proposing a synergistic effect between nucleotides and glutamate [6,7]. According to the multidimensional scaling experiment by Yamaguchi, the four basic tastes (sweet, sour, salty, and bitter) are placed at the vertices of a tetrahedron, and the umami taste is distant from any vertex [8]. Ninomiya group found that single fibers in mouse glossopharyngeal nerves responded favorably to monosodium glutamate (MSG) while hardly perceiving NaCl [9]. Kim et al. compared and measured the intracellular Ca^2+^ flow signal after salicin and umami peptides acted on bitter taste receptors, finding that five representative umami peptides in soybean could significantly dilute bitter taste [10]. From the flavor scoring test by Arai, the umami in peptides is mainly caused by the high molar content of glutamate and hydrophilic amino acids [7]. Andre et al. found that Glu/Asp-X peptides containing terminal acidic residues exhibited umami properties measured by tongue sensing [11]. Nakamura added the NaCl-response inhibitor amiloride to a mixture of MSG and 5′-GMP and found that it did not affect the umami response caused by their synergistic effect, indicating umami is an independent taste distinct from salty [12]. With the structure of metabotropic glutamate receptor 1/4 (mGluR1/4) and taste receptor type 1 member 1/3 (T1R1/T1R3) revealed, umami is widely accepted as a fundamental taste [13,14]. There have been many studies on umami dipeptides, mainly focusing on sequence identification, isolation and purification, participation in the Maillard reaction, and so on. Nevertheless, a series of crucial scientific issues such as action mechanism at the atomic level, structure–activity relationship, and molecular modification of umami dipeptides remain unclear.

According to the intracellular signaling pathway of umami perception [15,16], the T1R1/T1R3 will be activated upon binding to umami peptides (see Figure 1C). The dissociated G_β/γ_ subunit interacts with the phospholipase C-β2 to produce diacylglycerol (DAG) and inositol 1,4,5-triphosphate (IP3) by cleaving phosphatidylinositol 4,5-bisphosphate (PIP2). Subsequently, IP3 activates both the type III IP3 receptor (IP3-R) and the endoplasmic reticulum (ER), accompanied by the release of Ca^2+^ ions. It transiently activates transient receptor potential cation channel subfamily M member 5 (TRPM5), leading to membrane depolarization. As compensation for Ca^2+^ outflow, the influx of Na^+^ triggers the opening of ATP channels—pannexin-1 (Px-1) and calcium homeostasis modulator 1 (CALHM1)—releasing the neurotransmitter ATP. Finally, the afferent nerve fibers are stimulated to complete the signal transduction of taste receptor cells. By observing licking behavior in mice, Yamaguchi group investigated the effects of oxidized arachidonic acid (AA) and hexanal on bitter/umami perception [17]. They also found that MSG can activate G protein-coupled receptors (GPCRs) through G protein effectors, second messengers, and signal transduction pathways, resulting in the release of neurotransmitters from taste receptor cells. Using heterologous expression and calcium imaging strategies, Nelson et al. found that umami stimulation can activate T1R1/T1R3, releasing G_β/γ_ subunits and then regulating cAMP levels [18]. Using immunohistochemical experiments in mice, Damak et al. found that there appear to be TRPM5-dependent and -independent pathways for the identification of bitter, sweet, and umami tastes, where G_β/γ_ is crucial for umami taste transduction [19]. Through intracellular calcium assay, Liu et al. found that the binding of IP3 to IP3R induces the release of Ca^2+^ from the endoplasmic reticulum [20].

Based on structural and functional differences, GPCRs can be divided into four classes. Specifically, class A (i.e., rhodopsin type) includes visual receptors (such as rhodopsin), adrenergic receptors (such as β-adrenergic receptors), dopamine receptors, etc. Class B (i.e., secretin type) is mainly composed of numerous hormone receptors, such as adenylate A2A/A2B/A1/A3 receptors, glucagon receptors, and growth hormone-releasing hormone receptor (GHRHR), etc. Class C (i.e., glutamate type) includes some neurotransmitter receptors, such as the C5a receptor, chemokine receptor, lysophosphatidic acid (LPA), mGluR1, T1R1/T1R3, calcium channels, etc. Class D (i.e., the Frizzled/Smoothened type) includes receptors involved in development and cell signaling, such as smooth muscle cell receptors P2Y, Frizzled receptor, Hedgehog receptor, etc.

A series of umami receptors have been reported, which belong to the GPCR transmembrane proteins with a similar structure. The structure comprises an N-terminal Venus flytrap (VFT) domain and a 7-transmembrane domain (7-TMD), as well as a cysteine-rich domain (CRD) that connects the VFT and 7-TMD domains. In 1996, Chaudhari et al. identified the first taste-sensing metabotropic glutamate receptor 4 (mGluR4) by reverse transcription–polymerase chain reaction (RT-PCR) [21]. Through neural recording and immunoprecipitation reaction experiments, Nelson reported the existence of T1R1/T1R3 heterodimers [18]. In addition, Toyono determined brain-mGluR4 and brain-mGluR1 by immune tracing method, and then San Gabriel obtained taste-sensing mGluR1 through cloning and sequence analysis [22,23]. Wellendorph et al. identified group 6 subtype A of the class C GPCRs (GPRC6A) [24]. Bystrova et al. successfully identified the calcium-sensing receptor (CaSR) by analyzing RNA transcripts [25]. Haid et al. revealed the rhodopsin-like class A GPCRs (GPR92) through immunohistochemistry and cell quantification experiments [26]. It has also been reported that L-Glu binds tightly to the T1R1-VFT hinge region, while 5′ -nucleotides such as GMP and IMP are inclined to bind the T1R1-VFT open region, further stabilizing the closed conformation of T1R1 [27,28]. It has been demonstrated that the 7-TMD of T1R1 is the binding site for the umami compound N-(heptane-4-yl) benzo[d][1,3]dioxo-5-carboxamide [27]. As the common subunit for T1R1/T1R3 and the sweet taste receptors T1R2/T1R3, the 7-TMD of T1R3 is also the binding site for sweet substances such as lactisole and cyclamate. Notably, the umami receptor T1R1/T1R3 can respond directly to the stimulation of lactisole and cyclamate above, with the former inhibiting the activity of T1R1/T1R3 and the latter enhancing the sensitivity to L-Glu [29]. In sum, it is widely accepted that both T1R1- and T1R3-VFT may be potential binding sites for umami dipeptides [30,31,32].

There is no doubt that the synergistic interactions between glutamate and nucleotides can enhance umami taste perception. Since TIR1/T1R3 is the only umami receptor sensitive to both glutamate and nucleotides, it has become the research focus on the umami mechanism and dipeptide recognition [33]. Thus far, the 3D structure of human T1R1/T1R3 has remained unresolved, significantly impeding the exploration of the recognition mechanism by umami dipeptides at the atomic level. Cascales and Deng et al. constructed a complex model of L-Glu with T1R1-T1R3 based on the metabotropic mGluR (PDB ID: 1EWK) [34]. Molecular dynamics (MD) simulation results indicate that with the binding of L-Glu, the recognition pocket of T1R1 will experience a certain closure motion; Yu et al. established a significant correlation between the structure and umami intensity for a series of functional peptides [35]. Studies on umami synergy have demonstrated that the binding of MSG can enhance the recognition of umami peptides by T1R1/T1R3 receptor [32]. As shown in Figure 1D, the binding of inosine monophosphate (IMP) to the T1R1 hinge region promotes the new recognition of MSG by another neighboring molecule [27].

As a typical heterodimer, both subunits of T1R1/T1R3 possess similar structural components—VFT, CRD and 7-TMD domains. They are responsible for signal recognition, auxiliary ligand interactions, and signal transduction, as well as conformational changes and signaling transmembrane transmission (see Figure 1D). The three conserved structural domains work synergistically to enable the membrane receptor to accurately sense and transmit external signals into the cell interior. As a possible binding site for umami dipeptides in T1R1 and T1R3, VFT plays a key role in regulating the activity of GPCR receptors. In the inactive T1R1/T1R3 system, the VFT opening closes with the activation site being blocked. As the receptor T1R1/T1R3 binds to an active ligand, the VFT opening opens up accompanied by exposure of the active site, thus activating the downstream signaling pathway. The purpose of this work is to investigate the binding mode of umami dipeptides with T1R1/T1R3 VFT and to provide feasible suggestions for the possible optimization of peptide structure.

In this work, the human T1R1/T1R3 amino acid sequence was first determined from the UniProt database. Next, homology modeling was performed using SWISS-MODEL to obtain a three-dimensional model of T1R1/T1R3, referring to mGluR1 (PDB ID: 1EWK) [4] as a structural template. Here, what we use to construct the T1R1/T1R3 receptor model is the extracellular ligand binding domain part of mGluR1. Moreover, a personalized dipeptide database with a total of 400 samples was generated by utilizing the StoneMIND Collector tool for subsequent virtual screening. Next, complex models of the T1R1/T1R3 umami receptor with the screened dipeptides were established with molecular docking experiments. And then, comparative molecular dynamics (MD) simulations were carried out to explore the umami mechanism of dipeptides. Finally, the potential umami dipeptides were optimized with semi-rational design considering hydrophilicity and volume factors, with conformational mining criteria of the minimum binding free energy.

## 2. Results and Discussion

### 2.1. Construction of the T1R1/T1R3 Model

#### 2.1.1. Moderate Sequence Similarity to the Template

Prior to structural modeling, the sequence of human T1R1/T1R3 was first aligned with that of the template protein mGluR1, a key receptor regulating excitatory synaptic transmission in the central nervous system (CNS). Based on the crystal structure of mGluR1, Zhang et al. conducted mutagenic experiments on human T1R1 VFT, proving that the two receptors have similar receptor–ligand binding mechanisms [27]. As shown in Figure S1, the sequence similarity/consistency of T1R1 and T1R3, respectively, with the mGluR1 crystal structure (PDB ID: 1EWK) is 44%/26% and 40%/25%, which is in line with previous bioinformatics studies by Cascales and Dang [34]. It can also be observed that the regions with high sequence agreement are distributed randomly, not selectively located in the expected substrate recognition pocket.

#### 2.1.2. High Structural Similarity to the Template

**Figure 2:**
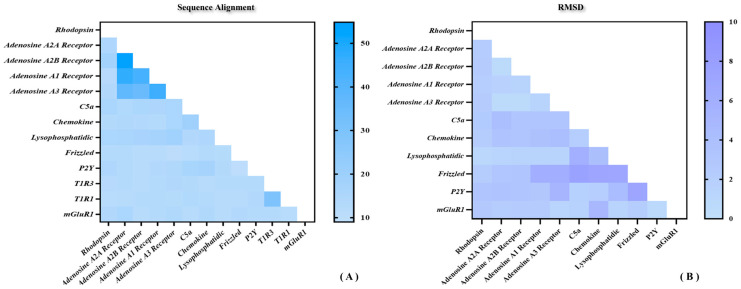
Sequence and structural similarity of representative receptors for the four classes of GPCRs. (**A**) Sequence alignment statistics of a total of 13 representative GPCRs including T1R1/T1R3. (**B**) Conformational RMSD statistics for a total of 11 representative GPCRs.

As shown above, the sequence identity and similarity between the umami peptide receptor T1R1/T1R3 and the template mGluR1 are not as high as expected. Does this imply that the results of homologous modeling may be doubtful? Figure 2 shows the sequence and structural similarity of representative receptors for the four classes of GPCRs. As shown in Figure 2A, the highest and lowest sequence identity was only 45% and 10%. It suggests a generally moderate sequence identity between different GPCRs, naturally including the template mGluR1 and T1R1/T1R3. By superimposing the 11 representative GPCRs structures above, 45 root mean square deviation (RMSD) values were obtained with an average of about 2 Å (see Figure 2B). In fact, this degree of conformational deviation could be overcome by subsequent MD simulations. In sum, despite the medium sequence identity of T1R1/T1R3 with mGluR1, the high structural similarity between GPCRs is enough to ensure homology modeling reliability for the umami receptor.

#### 2.1.3. Rationality Evaluation of the T1R1/T1R3 Modeled Structure

**Figure 3:**
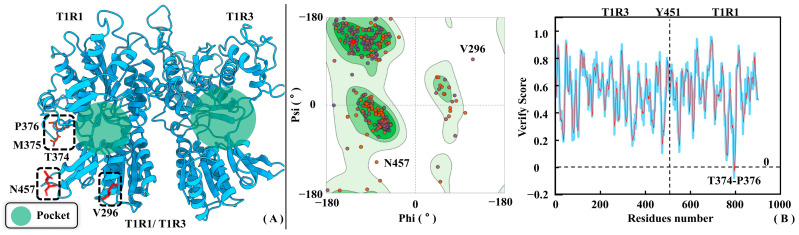
Rationality assessment of T1R1/T1R3 homology modeling results. (**A**) Three-dimensional structure of the T1R1/T1R3 model. (**B**) Ramachandran distribution (left panel) and Profile-3D (right panel) strategies were both used to assess the rationality of the modeling structure. In the Ramachandran plot, green, light green, and white colors indicate the optimal, allowed, and disallowed zones, respectively.

Figure 3 shows the homology modeling results for T1R1/T1R3, where Ramachandran plots and Profile-3D distributions were both employed to assess its rationality. According to the Ramachandran plot, the proportions of side-chain dihedral angles of T1R1/T1R3 falling in the conformationally optimal and allowed zones are 87.7% and 10%, respectively. Only V296 and N457 fall in the disallowed region, fortunately both far away from the substrate pocket of T1R1/T1R3 (see Figure 3A). This fully proves the modeled conformation being in accordance with the statistical geometric rules. According to the Profile-3D analysis, the verified score and verified expected high score of the model are 354.57 and 373.24, respectively. In addition, the verify expected low score of 136.053 is much lower than the former two, which fully demonstrates the high rationality of the T1R1/T1R3 modeled structures. As shown in Figure 3B, only T374, M375, and P376 in the T1R1/T1R3 model possess verified scores less than 0. Like V296 and N457 previously mined from the Ramachandran plot, T374-P376 of T1R1 is also far away from the substrate binding pocket, having little impact in subsequent studies on conformational changes and molecular recognition.

### 2.2. Determination of Representative Umami Dipeptides

#### 2.2.1. Construction of a Dipeptide Structure Database

The dipeptide structure database is composed of 400 samples, with items including sequence, 3D structure, physicochemical parameters (such as molecular weight/isoelectric point/hydrophobic coefficient), and umami prediction probability (see dipeptide-database.xlsx). In light of the following series of research backgrounds, the isoelectric points and hydrophobicity coefficients of dipeptides were both calculated and imported into the database. Arai et al. found that bitterness and umami inhibit each other, and hydrophobicity is one of the key factors causing bitterness [36]. Beksan et al. synthesized glutamic acid glycoconjugates using the Maillard method, showing a strong umami taste [37]. Zhang et al. determined that tomato seed powder could be thoroughly hydrolyzed to obtain umami amino acids with the highest efficiency at a pH value of 3 [38]. According to the supplementary file dipeptide database.xlsx, WW (Trp-Trp) and GG (Gly-Gly) dipeptides, respectively, possess the maximum and minimum molecular weights (MWs). Acidic and basic dipeptides are predictably widely distributed. The lowest and highest isoelectric points (PI) of the dipeptides were 3.75 and 11.31, respectively. The net charge of the dipeptide ranged from −2.02 to 6.11 at pH value of 7. The dipeptides also showed a wide range of hydrophobicity, with AA (Ala-Ala, ~4.45) and EE (Glu-Glu, ~−4.1) having the largest and smallest ones, respectively.

**Figure 4:**
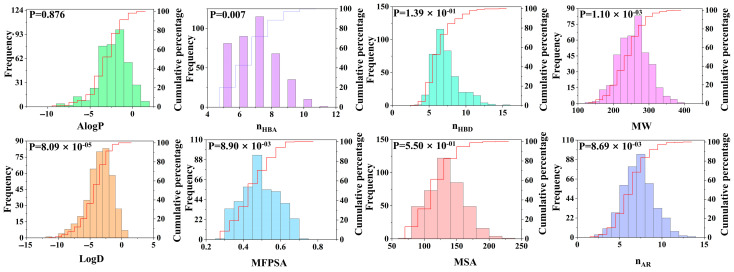
Distribution of 8 molecular properties of dipeptide compounds, including LogD, AlogP, MW, nHBD, nHBA, nAR, MFPSA, and MSA.

To better analyze the characteristics of the dipeptide database, we introduced molecular descriptors (an important measurement tool) to characterize the molecular properties. Compounds with similar biological activities typically exhibit similar molecular descriptor characteristics. This study focused on eight molecular properties, namely AlogP, nHBA, nHBD, MW, LogD, MFPSA, MSA, and nAR, and analyzed the intrinsic relationships between these properties and dipeptide compounds (see Figure 4). Among the eight molecular properties studied, two properties of LogD and AlogP are closely related to the hydrophobicity of the molecules. The distribution range of LogD was −11.59 to 0.88, and the average value was −3.50. The value range of AlogP was −8.93 to 1.56, and the average value was −2.31. These data indicate that dipeptide compounds exhibit relatively high hydrophilicity, which makes them more likely to bind to hydrophilic receptor pockets than other compounds. Further analysis revealed that at the 95% confidence level, the correlated *p* value of the AlogP difference of the compound was 0.876, which was significantly greater than the *p* value of LogD (8.09 × 10^−5^). This indicates that there are significant differences in the distribution between LogD and AlogP, and that LogD has a more prominent classification ability for umami dipeptides.

#### 2.2.2. Screening Strategies for Umami Dipeptides

Table S1 lists the physicochemical parameters, binding energies, and umami prediction probabilities for 33 experimentally reported umami dipeptides. In general, the receptor–ligand binding free energy is less than −6.5 kcal·mol^−1^ which corresponds to a dissociation constant of around 10^−5^ M, indicating a relatively strong affinity. To verify the rationality of the docking, we also adopted Glide (4.12.0) and conducted secondary docking. The docking scoring results are presented in parentheses b and c in Table S1. The results show that whether AutoDock Vina or Glide is adopted, the docking energy of DG (Asp-Gly) and EK (Glu-Lys) performs excellently among the 33 dipeptides, indicating that it is reasonable and reliable for us to choose these two dipeptides for subsequent research. Among the 33 umami dipeptides, 30 samples (~90.9%) possess binding energies of less than −6.5 kcal·mol^−1^ to T1R1 pockets, and it was also found in 16 samples (~48.5%) to T1R3 pockets, and 15 samples (~45.5%) to both T1R1 and T1R3 pockets. According to the data of PI and hydrophobicity, a total of 26 dipeptides (~78.8%) are both acidic and hydrophilic. The umami perception probability in 22 dipeptides (~66.7%) exceeds 85%, which indicates that the prediction method of the UMPred-FRL server is relatively reliable. Referring to the supplementary file dipeptide-database.xlsx, the ratio of samples positively found for 33 umami dipeptides to the other 367 samples was 0.89:0.35, which fully proves the accuracy of the umami prediction. There were 15 dipeptides with binding free energies less than −6.5 kcal·mol^−1^ to both pockets of T1R1/T1R3, but only 11 samples (~73.3%) were detected with an umami prediction probability greater than 85%.

In sum, the screening of umami dipeptides should comprehensively consider various factors such as the binding free energy, probability of prediction, PI value, hydrophobicity, and emphasis on D/E residues [7]. In this work, the DG with a smaller molecular weight and volume, and the EK with a larger parameter were both selected as representative ligands to explore molecular recognition with receptor–ligand interaction, conformational changes, and the subsequent optimization of umami dipeptides.

### 2.3. Binding Modes of Umami Dipeptides EK and DG to Receptors T1R1/T1R3

**Figure 5:**
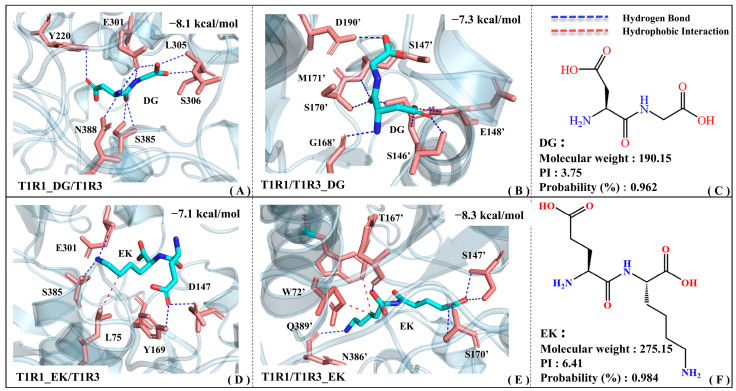
The complex models of T1R1/T1R3 with the umami dipeptides DG and EK. Binding modes of the T1R1_DG/T1R3 (**A**), T1R1/T1R3_DG (**B**), T1R1_EK/T1R3 (**D**), and T1R1/T1R3_EK (**E**) systems, where blue and red dashed lines are used to denote the hydrogen bonding and hydrophobic interactions, respectively. The 2D structure and physicochemical parameters for the DG (**C**) and EK (**F**) peptides.

Figure 5 shows the binding patterns of the screened umami dipeptides (i.e., EK and DG) with T1R1 and T1R3, respectively. To better distinguish the residues in T1R1 and T1R3, an apostrophe is added to the upper right corner of the latter, while the former remains unlabeled. In the T1R1_DG/T1R3 complex model, the Y220/E301/L305/S306/S385/N388 residues form relatively stable hydrogen bonds with the DG dipeptide. EK not only forms hydrogen bonds with E301/S385/D147/Y169, but also hydrophobic interactions with L75 in the T1R1 binding pocket (see Figure 5A,D). Although EK is larger than DG, it has relatively fewer contact residues in T1R1, suggesting a relatively weak molecular recognition. According to molecular recognition exploration by Cascales, E301 and S148 (an adjacent residue to D147) in T1R1 are both key residues for recognition by the umami glutamate [34]. Based on quantum-mechanical (QM) and molecular mechanics (MM) analyses, Wang et al. found that L305 and S306 both contribute to complex formation between T1R1 and the umami peptide DF9 (DVILPVPAF) [39]. Kniazeff et al. demonstrated that Y222 is critical for the activation of metabolized glutamate receptors (mGluRs) based on site mutation experiments. Y222 is actually only about 3.5 Å away from the T1R1 pocket residue Y220 provided above, which also supports the high confidence of the binding position for the DG dipeptide [40]. With reference to the molecular docking results of a series of umami peptides from myosin, E301/Y220/D147 appeared frequently at the recognition interface of the complex model [31]. According to the molecular modeling data of Liu and Wang et al., S385/N388 of T1R1 is the potential binding site for the beefy meaty peptide (BMP) [30,41]. According to fluorescence resonance energy transfer (FRET) and immunoprecipitation experiments [42], Nuemket’s group proposed that S594 was highly conserved in T1R1 and class C GPCRs, only being around 3.5 Å away from the pocket residues Y220/Y169 above. Using a multi-color fluorescence spectral energy transfer (MFSEC) strategy, they also demonstrated that D288/D289 leads to the closure of the T1R2a VFT gap through water-mediated hydrogen bonding. It is speculated that the neighboring D147 also has a high probability of forming hydrogen bonds with the large umami peptide EK, and then undergoing similar motion patterns.

In the T1R1/T1R3_DG complex model (see Figure 5B), DG forms stable hydrogen bonds with the S146′/S147′/G168′/S170′/M171′/D190′ residues. The relatively larger umami dipeptide EK forms hydrogen bonds with S147′/S170′/N386′/Q389′ in the T1R3 pocket, and also possesses hydrophobic interactions with W72′/T167′ (see Figure 5E). According to the isolation and characterization experiments of umami enhancement peptides from *Ruditapes Philippinarum*, the S147′/T167′/S170′ residues are involved in molecular recognition by peptides [43]. Zhu et al. extracted novel umami peptides from Chinese anchovy sauce and found that E148′ (adjacent to S146′/S147′) of T1R3 shows certain umami effects and might be one of the key residues for receptor–ligand recognition [44]. Based on the structural basis of T1R taste receptors perceiving different chemicals and sequence comparison, the S146′/D190′/Q389′ residues of T1R3 may interact with non-specific amino acids [42]. Walters et al. constructed a T1R3 homology model, demonstrating that N74′ (corresponding to N386′ of T1R3) is involved in binding to neotame [45]. According to the taste mechanism exploration of umami peptides from Chinese traditional fermented fish (i.e., Chouguiyu), umami dipeptides including EE and EV tend to form hydrogen bonds with residues such as Ser/Glu/Gly/Met/Trp in T1R3, which is exactly in agreement with the residue groups reported above [46].

To sum up, by mining the above key residues and comparing with previous studies, the reliability of a binding pattern of T1R1/T1R3 with DG and EK is thoroughly proved. Notably, the key residues extracted in this work are not completely in line with the data reported previously, which might be associated with the sequence alignment and homology modeling errors of T1R1/T1R3. It should be noted that the binding energy of DG with the T1R1 pocket (−8.1 kcal·mol^−1^) is lower than that in the T1R3 pocket (−7.3 kcal·mol^−1^), whereas it is reversed for EK (−7.1 vs. −8.3 kcal·mol^−1^). In addition, EK can establish strong hydrophobic interactions with the pockets of both T1R1 and T1R3, which is related to its large geometric volume.

### 2.4. Pocket Characteristics of T1R1/T1R3

Based on the binding patterns above, DG and EK possess a certain binding preference for the T1R1/T1R3 pocket, with the former for T1R1 and the latter for T1R3. To unveil these binding differences in the representative umami dipeptides DG/EK, it is essential to provide the pocket characteristics including both the volume and charge environment of T1R1/T1R3.

#### 2.4.1. The Pocket Size of T1R3 Is Significantly Larger than That of T1R1, Conditionally Incorporating Bigger Substrates

The geometric data of length, area, and volume for the T1R1/T1R3 pockets and the DG/EK peptides are both compared. As depicted in Figure S2A, the T1R1 pocket presents a closed state as a whole, with a length/width/height of 13.26/9.53/6.27 Å and a volume of 1070.20 Å^3^. The T1R3 pocket generally remains open, with corresponding sizes of 16.07/11.37/8.67 Å and 2374.46 Å^3^. For the representative umami dipeptides DG and EK, the maximum lateral/longitudinal distances and space volume of the former were 8.30/7.20 Å and 306.24 Å^3^, with the corresponding data of the latter being 13.10/8.20 Å and 482.60 Å^3^. Through crystallography experiments by Nuemket, it is known that there is a binding pocket with a length/width/height of 16/12/7 Å between T1R2a and the L-Glu ligand, which is not completely isolated from the solvent. They also measured the ligand binding space of another homologous mGluR, with a size of 11.5/10.5/7 Å [47]. Similar pocket sizes not only confirmed the substrate recognition sites for T1R1/T1R3, but also showed that the T1R3 pocket was significantly larger than T1R1, being suitable for binding larger umami dipeptides. In fact, it was also consistent with a previous comparison of binding energy (see Table S1).

#### 2.4.2. The T1R3 Pocket Is Generally Alkaline with a Larger Channel Radius

**Figure 6:**
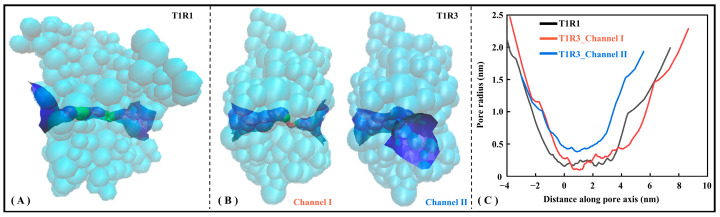
Hole analysis of T1R1/T1R3 pockets. Pockets of T1R1 (**A**) and T1R3 (**B**) are shown, respectively, with the latter containing channels I and II. The stereoscopic view of the channel where the green and blue pores allow substrate to pass through, while the red one is too small in radius to accommodate even a single water molecule. (**C**) Axial changes of the three-channel radius.

To present a more intuitive view of the pocket microscopic features, the Hole program [48] was employed to visualize substrate channels for T1R1/T1R3. Figure 6 shows the radius axial distribution of substrate channels for the T1R1 and T1R3 systems. Specifically, T1R1 has one substrate channel traversing horizontally (see Figure 6A), with T1R3 having both horizontal (i.e., Channel I) and vertical (i.e., Channel II) channels (see Figure 6B). As shown in Figure 6C, the pore radius near the starting point in the T1R1 molecular channel maintains only 0.16 nm, indicating that it is difficult to bind and transport bulky substrates. Nevertheless, it is avoided in T1R3 with two substrate channels, whose average radius values are 0.84 and 0.72 nm, respectively. Here, irregular concentric circles are drawn along the central axis at a spacing of 0.25 Å, with the two nearest contact residues being adopted to characterize the channel details.

Table S2 lists all the contact residues on three channels of T1R1/T1R3. It can be observed that a large number of contact residues located in T1R1 substrate binding pocket (i.e., L305, S385, E301, D147, Y220, L75, S306, and Y169) appear around the molecular channel, with the similar features being found in the T1R3 substrate binding pocket (i.e., D190′, N386′, Q389′, S170′, E148′, S146′, S147′, T167′, and G168′). All other pocket residues lie within a 2 Å range of the channels, fully indicating that the three channels of T1R1/T1R3 pass right through its two substrate binding pockets.

To further analyze the acid–base preference of the molecular channel, Propka3 is used to calculate the pKa values for titratable residues. The calculation of pKa not only considers the environmental pH value, but also fully involves influence factors including the solvent, Coulomb force, hydrogen bond, and electrostatic potential. Due to the multi-factorial weighting and assignment, the calculated pKa values are slightly outside the conventional range of −2 to 14 [49]. As listed in Table S3, the average pKa at the residue level in descending order is T1R3_channel I (~8.74), T1R3_channel II (~8.57), and T1R1_channel (~7.51). Molecular channels of T1R3 and T1R1 are generally basic with the latter being relatively higher, which is conducive to binding acidic substrates. This is in excellent agreement with the conclusion that umami peptides are generally acidic, such as the PI values of EK and DG being 6.41 and 3.71, respectively. From the perspective of substrate delivery, it is speculated that the weakly alkaline T1R1 channel may be more easily recognized by the moderately acidic umami dipeptide DG, as well as T1R3 channels by EK.

### 2.5. Molecular Dynamics Simulation of Umami Dipeptide Recognition with T1R1/T1R3

#### 2.5.1. Trajectory Convergence Is a Prerequisite for Molecular Recognition and Conformational Change Analyses

To investigate the molecular recognition of EK and DG by the T1R1/T1R3 pockets, respectively, comparative MD simulations were performed for the T1R1_DG/T1R3, T1R1/T1R3_DG, T1R1_EK/T1R3, T1R1/T1R3_EK, and T1R1/T1R3 systems. The simulation process was repeated three times. The results of the three experiments were well repeated, and the data were reasonable and reliable. Therefore, one set of data was used for the subsequent system analysis. The mean and standard deviation of potential energies for the five systems above are about −2.35 × 10^5^ and 0.01 × 10^5^ kcal·mol^−1^, which indicates that the MD simulations are smooth with conformational sampling being more reasonable. In addition, their time-dependent root mean standard deviation (RMSD) distribution is generally stable, converging within 0.50–0.66 nm, which is slightly higher than that of conventional crystal structures (~0.2 nm), being related to the error of the homologous modeling model of T1R1/T1R3.

Figure S3A shows the root mean square fluctuation (RMSF) distribution at the residue level for the five systems above. Among all the systems, T1R1 S172 and T1R3 L99′ both possess relatively low RMSF values of approximately 0.25 nm and 0.15 nm, respectively. This suggests that the two residues may be located at the hydrophobic core and serve as structural supports. In addition, with the binding of the umami dipeptide DG, the flexibility of T1R1 V211 and T1R3 K86 was significantly improved synergistically. According to the reverse transcription–PCR (RT-PCR) experiments by Yoshida, the umami synergistic mechanism of MSG stems from allosteric regulation [50]. That is, the binding of MSG triggers spatial conformational changes of the umami receptor. As shown in Figure S3B, four complex models (i.e., T1R1_DG/T1R3, T1R1/T1R3_DG, T1R1_EK/T1R3, and T1R1/T1R3_EK) exhibit high RMSF correlations with the T1R1/T1R3 system, respectively, with the R-value and k-slope being 0.77/0.80/0.74/0.67 and 0.66/0.93/0.68/0.72, respectively. To sum up, the self-consistent flexibility distribution proves the rationality of MD trajectories, which is a prerequisite for the subsequent analyses of pocket features and molecular recognition.

#### 2.5.2. The Binding of Umami Peptides Results in Conformational Closure of VFT

**Figure 7:**
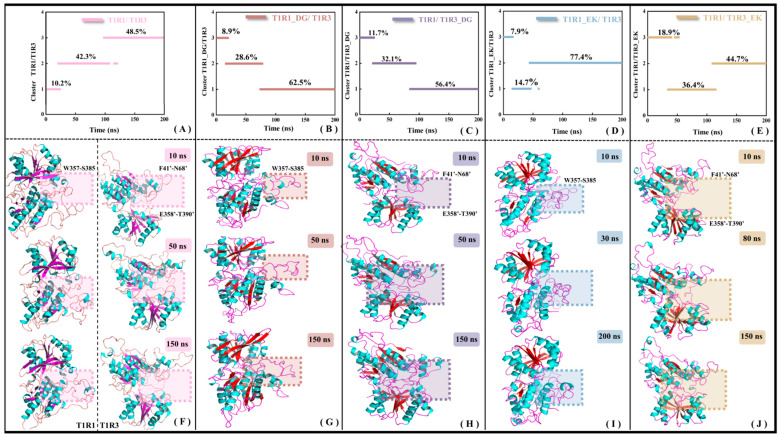
Conformational clustering (**A**–**E**) and representative conformational superimposition (**F**–**J**) of MD trajectories for the T1R1/T1R3 (**A**,**F**), T1R1_DG/T1R3 (**B**,**G**), T1R1/T1R3_DG (**C**,**H**), T1R1_EK/T1R3 (**D**,**I**), and T1R1/T1R3_EK (**E**,**J**) systems. The boxed sections in panels F-G provide the significant allosteric features. The β-folds in the ligand-free T1R1/T1R3 in panel F are indicated in purple, with that in the ligand-bound one in red.

As a core member of the class C GPCRs, the full-length structure of the umami receptor is mainly composed of three domains, i.e., VFT, CRD, and 7-TMD. The VFT domain is responsible for signal capture, the CRD domain is involved in signal transduction, and the 7-TMD domain plays a key role in signal transmembrane transmission. The research object in this work is the extra-membrane VFT of T1R1/T1R3, which is still denoted by T1R1/T1R3 for convenience. The previous RMSF analysis (see Figure S3) has shown that the overall molecular flexibility of T1R1/T1R3 decreases with the binding of DG or EK, accompanied by co-allosteric flexibility of the residues. To understand the details of conformational change, clustering analyses were performed for the MD trajectories of the T1R1/T1R3, T1R1_DG/T1R3_DG, T1R1_EK/T1R3, and T1R1/T1R3_EK systems. Considering that the investigated systems were derived from homology modeling, as well as the trajectory RMSD being distributed in the range of 0.50–0.66 nm, the criterion threshold of conformation clustering was set at 0.5 nm. As shown in Figure 7A–E, the trajectories of the five systems are, respectively, divided into three clusters, with certain conformational transitions between them.

Superimposition of the lowest energy conformation from each cluster (i.e., representative conformation) helps to explain the time-dependent allosteric details of the system. Compared to T1R1/T1R3 (see the left panel of Figure 7F), the opening formed by the T1R1 pocket loop (W357–S385) in T1R1_DG/T1R3 gradually closes (see Figure 7G), and the similar change for the umami receptor T1R1 after Glu binding, Cascales found that pocket loop in the active T1R1 is progressively closed. If it maintains a persistently open state, the substrate Glu will experience diffusion and thus be away from the binding pocket [34]. In the MD simulations of T1R1/T1R3_DG (see Figure 7H) and T1R1/T1R3_EK (see Figure 7J) systems, two loops F41′–N68′ and E358′–T390′ of T1R3 exhibit conformational closure, facilitating the association of substrates DG and EK. The latter has a relatively small allosteric amplitude, which may be related to the large volume of the substrate EK. Based on fluorescence-detection size-exclusion chromatography (FSEC) [51] and structural biology experiments on bacterial periplasmic binding protein [52], with mGluR binding by umami substances, CRD undergoes displacement resulting in the conformational closure of VFT, and then transfers the umami signal to 7-TMD, leading to activation of the downstream transmembrane region and cytoplasmic G protein. In addition, the crystal structure of the VFT dimer in the inactive and active (complexed with the substrate Glu) states tends to be in the open and closed states, respectively [40], which is highly consistent with the loop closure motion observed for T1R1/T1R3 recognized by the DG or EK umami dipeptides (see Figure 7F–J).

#### 2.5.3. The Hydrophilic Pocket Facilitates the Binding of Umami Dipeptides

Whether the receptor pocket is accommodated by water molecules is an important scientific issue that has attracted much attention in the field of functional molecular design. Generally, deep hydrophobic cavities have a high probability of forming active pockets, among which the replacement and introduction of non-polar groups should be emphasized in the subsequent molecular design. Polar water molecules are also commonly present in active pockets, and often play a key role in maintaining stable receptor–ligand binding. In drug design based on a hydrophilic pocket structure, the compound’s molecular affinity, selectivity, and pharmacokinetic properties can be improved by replacing pocket water molecules or designing water-mediated hydrogen bonds [53,54].

**Figure 8:**
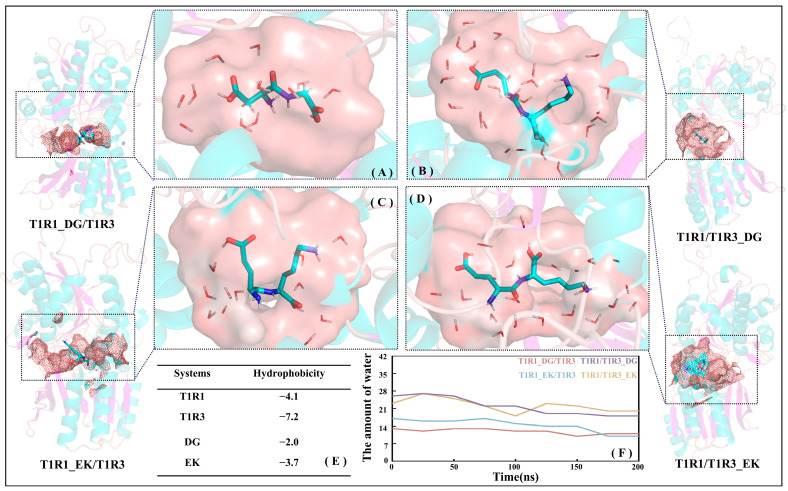
Hydrophilic pockets of T1R1 (**A**,**C**) and T1R3 (**B**,**D**) both contribute to the association with the DG (**A**,**B**) or EK (**C**,**D**) umami dipeptides. The hydrophobicity of the T1R1/T1R3 and DG/EK (**E**) systems as well as time-dependent variations for the number of ambient water molecules in the pockets of the four complex systems (**F**).

Based on the MD trajectories for the T1R1_DG/T1R3 (see Figure 8A), T1R1_EK/T1R3 (see Figure 8C), T1R1/T1R3_DG (see Figure 8B), and T1R1/T1R3_EK (see Figure 8D) systems, the total hydrophobicity values of T1R1/T1R3 pockets were calculated using the Calculate Solvent Accessibility module embedded in Discovery Studio 3.5. The larger the positive value, the stronger the hydrophobicity, while the negative value indicates hydrophilicity. As shown in Figure 8E, the hydrophobicity values for T1R1/T1R3 and DG/EK are −4.1/−7.2 and −2.0/−3.7, respectively, indicating that the hydrophilicity of T1R3/EK is significantly stronger than that of T1R1/DG. Figure 8F shows the variation in the number of pocket water molecules with time during MD simulations. With the advance of MD simulations, the total number of water molecules in the T1R1_DG/T1R3, T1R1_EK/T1R3, T1R1/T1R3_DG, and T1R1/T1R3_EK pockets slowly decreases, finally converging to 13, 11, 18, and 19, which is consistent with the slow shrinking motion above (see Figure 7F–J). The T1R1 pocket holds fewer water molecules than the T1R3 pocket, which is closely related to the latter’s larger size and stronger hydrophilicity. The previous binding energy and size matching results (see Figure 4 and Figure S2) both show that EK and DG, respectively, tend to bind to T1R3 and T1R1, which can also be confirmed by the hydrophilicity order here (T1R3 > T1R1, EK > DG). According to the scoring experiment by Arai [7], the high umami perception is strongly correlated with the high molar content of hydrophilic Glu, which also suggests that the hydrophilic pockets of T1R1/T1R3 facilitate the binding of umami dipeptides.

#### 2.5.4. Stable Hydrogen Bonds Exist Between Umami Peptides and T1R1/T1R3

Hydrogen bonds play a key role in the interactions between small molecules and proteins, greatly affecting the stability, binding, and reaction processes of biological systems. Based on the MD equilibrium trajectories (150–200 ns) of the complex systems (i.e., T1R1_DG/T1R3, T1R1/T1R3_DG, T1R1_EK/T1R1, and T1R1/T1R3_EK), the changes in their intramolecular hydrogen bonds were compared. Geometric criteria are used to evaluate hydrogen bond formation [49], i.e., the spatial distance between the donor atom and acceptor atom is less than 3.5 Å, and the angle among the acceptor atom, hydrogen atom, and donor atom is greater than 135°.

As listed in Table S4, the total number of hydrogen bonds with more than 45% occupancy in the four systems (i.e., T1R1_DG/T1R3, T1R1/T1R3_DG, T1R1_EK/T1R1, and T1R1/T1R3_EK) is 9/5/7/10, respectively. It can be seen that there are more hydrogen bonds between DG and T1R1, while EK on the other hand prefers to bond with T1R3. Based on the 10/100/200 ns snapshots of the four complex systems, Figure S4 shows the key interactions between DG/EK and the T1R1/T1R3 pockets, with the hydrogen bond data being verified by Table S4 above. In addition to the T1R1 (i.e., S385/Y169/Y220/L75/S306/N388/E301/D147) and T1R3 pocket residues (i.e., D190′/S147′/E148′/G168′/T167′/S170′/S146′/Q389′), several channel residues including A302 also exhibit a high potential to form hydrogen bonds with dipeptides. According to molecular modeling and perception experiments by Yang, A302 stably binds to γ-glutamyl peptide via hydrogen bonds and hydrophobic interactions, contributing to its umami enhancement [55]. Using mass spectrometry (MS) to identify the performance of umami-enhancing peptides, Su found that His, as a bitter amino acid, is able to produce a balanced and delicious perception together with umami peptides [56]. By observing the T1R1/T1R3_DG and T1R1/T1R3_EK systems (see Figure S4D–F,J–L), the T1R3 pocket residue H145′ also coincidentally participated in the formation of a series of hydrogen bonds.

#### 2.5.5. Weak Interactions Favor the Recognition of Umami Peptides by T1R1/T1R3

**Figure 9:**
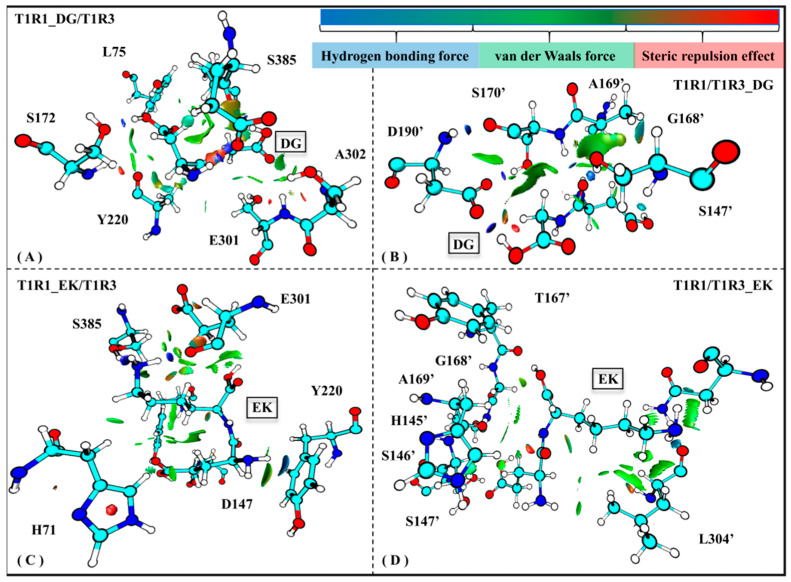
Weak interactions observed in the T1R1_DG/T1R3 (**A**), T1R1/T1R3_DG (**B**), T1R1_EK/T1R3 (**C**), and T1R1/T1R3_EK (**D**) systems. The magnitude of the force is indicated by different colors and areas on the RDG isosurfaces: the larger areas indicate stronger forces; blue, green, and red colors, respectively, indicate hydrogen bonds, van der Waals forces, and steric repulsion effects.

Weak interaction analysis based on electron density functionals can be widely employed to explore the local deformation of a biological system. Maruyoshi utilized this method to investigate conformational change of spermidine interacting with adenosine triphosphate in an aqueous solution [57]. To further explore the binding details of T1R1/T1R3 with the umami peptides DG/EK, Figure 9 shows the average weak receptor–ligand interactions for the four complex systems.

Based on the skeleton of the stable conformation, DG/EK is chosen as the graph center to draw the visual weak interactions. As shown in Figure 8A,C, the key residues E301/S385/A302/L75 in T1R1 form stronger van der Waals interactions, which was also mentioned in the previous analyses of the pocket binding mode (see Figure 5) and channel contact residues (see Table S2). Wang et al. used the LC-MS/MS method to identify and characterize novel umami peptides, demonstrating that L75 forms stable hydrogen bonds and hydrophobic interactions with T1R1 [58]. Similar interactions were also observed in the T1R3 pocket residues H145′, S147′, A169′, and D190′ (see Figure 8B,D). Like T1R1 L75, T1R3 T167′ was also able to form hydrogen bonds and van der Waals interactions with the dipeptide EK. In addition, S172 has a steric repulsion effect with the dipeptide DG, which also confirms its structural support role mentioned in previous RMSF analysis (see Figure S3A). In sum, in addition to hydrogen bonds, van der Waals forces and steric repulsion effects both aided the tight association of the umami dipeptides to the T1R1/T1R3 pocket.

### 2.6. Molecular Optimization of Umami Dipeptides Driven by Binding Free Energy

Common peptide optimization strategies include amino acid substitution, chain truncation, chain hybridization, and chain modification [59]. As a representative polar functional group, -OH groups can form hydrogen bonds with other O/N/F atoms, further increasing molecular polarity. In addition, chain length alteration may affect the conformational features of proteins, which is an essential factor strengthening non-polar interactions. Cao designed FFK/FYK/YYK/YFK short aromatic tripeptides by adding an -OH group to the benzene ring of phenylalanine, changing the hydrogen bonding force and self-assembly behavior of peptides [60]. With the lead compound hexapeptide being truncated to the antimicrobial tetrapeptide WRWR-NH2, Lau successfully obtained a potential therapeutic agent for the treatment of methicillin-sensitive Staphylococcus aureus (MSSA) and methicillin-resistant Staphylococcus aureus (MRSA) [61]. In addition, Berkov attached aliphatic chains at two different positions on the scaffold, and the anchoring efficiency of positively charged aminoglycosides on the bacterial membrane is greatly improved [62]. Based on the consideration of polar and non-polar interactions, novel potential umami dipeptides were designed, such as introducing the -OH group to DG/EK or changing the side-chain length, etc.

#### 2.6.1. Introduction of Hydroxyl Groups

**Figure 10:**
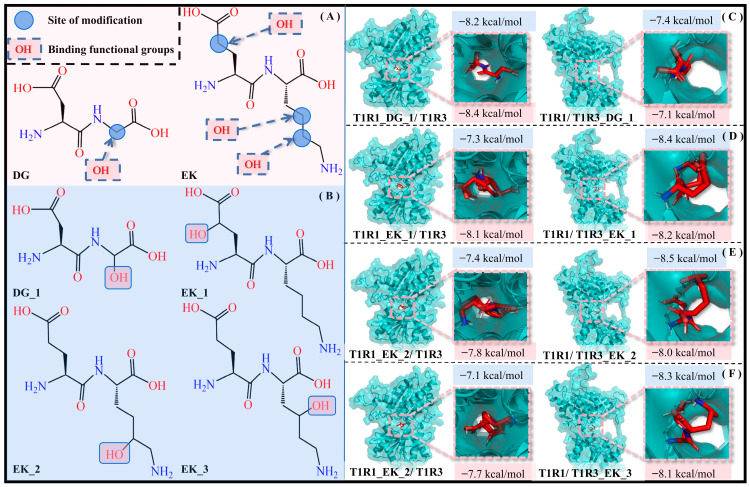
Introduction of -OH groups to umami dipeptides. (**A**) Structures and modification sites of the DG and EK umami dipeptides. (**B**) Molecular recognition of four new dipeptides—DG_1 (**C**), EK_1 (**D**), EK_2 (**E**), and EK_3 (**F**)—by the T1R1/T1R3 pockets, respectively. Blue and pink background data are the docking binding energy and SIE binding free energy, respectively.

According to the previous solvation effect exploration, the hydrophilic pocket facilitates the molecular recognition of umami dipeptides by T1R1/T1R3, and thus hydrophilic modification is an effective strategy to improve umami perception. By introducing an -OH group with strong hydrophilicity into the umami dipeptides DG and EK, it is expected to form hydrogen-bonding interactions with water molecules, thus enhancing their solubility and hydrophilicity. To rule out chance, the -OH group was respectively added to different carbon atoms in DG and EK, with four dipeptide derivatives (i.e., DG_1, EK_1, EK_2, and EK_3) being obtained (see Figure 10).

Eight complex systems (T1R1_DG_1/T1R3, T1R1/T1R3_DG_1, T1R1_EK_1/T1R3, T1R1/T1R3_EK_1, T1R1_EK_2/T1R3, T1R1/T1R3_EK_2, T1R1_EK_3/T1R3, and T1R1/T1R3_EK_3) were acquired by molecular docking experiments, and their docking energies were −8.2/−7.4/−7.3/−8.4/−7.4/−8.5/−7.1/−8.3 kcal·mol^−1^ are higher than those of the initial T1R1_DG/T1R3, T1R1/T1R3_DG, T1R1_EK/T1R3, and T1R1/T1R3_EK systems, corresponding to values of −8.1/−7.3/−7.1/−8.3 kcal·mol^−1^. Calculated by the SIE method, the binding free energies of the above eight complex models are −8.4/−7.1/−8.1/−8.2/−7.8/−8.0/−7.7/−8.1 kcal·mol^−1^, respectively. This not only demonstrated the effectiveness of introducing an -OH group in umami dipeptide modification, but also indicated the potential and feasibility of four dipeptide derivatives.

#### 2.6.2. Adjustment of Side-Chain Length

**Figure 11:**
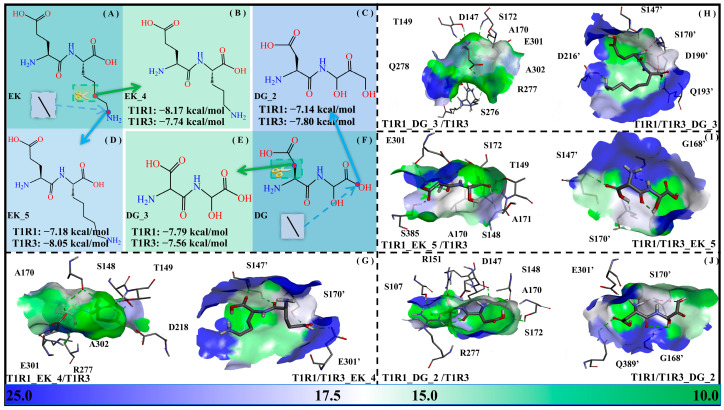
Adjustment of side-chain lengths of the umami dipeptides DG and EK. Molecular structures of the EK (**A**), EK_4 (**B**), DG_2 (**C**), EK_5 (**D**), DG_3 (**E**), and DG (**F**) systems, as well as their molecular recognition by the T1R1/T1R3 pockets (**G**–**J**). The data in panels (**B**–**E**) refer to binding free energies between modified dipeptides and T1R1/T1R3 pockets. In panels (**G**–**J**), blue-green bars are used to represent the solvent-accessible surface area of the T1R1/T1R3 pocket.

The solvent-accessible surface area (SASA) values indicate the solvent accessibility of a protein, which can be used to describe not only the solubility of the protein but also the molecular recognition between the acceptor and ligand. For representativeness, a methylene group was added/subtracted for EK and DG, respectively, with the dipeptide derivatives EK_5, EK_4, DG_2, and DG_3 being obtained. Their binding free energies with the T1R1/T1R3 pockets (i.e., T1R1_EK_5/T1R3, T1R1/T1R3_EK_5, T1R1_EK_4/T1R3, T1R1/T1R3_EK_4, T1R1_DG_2/T1R3, T1R1/T1R3_DG_2, T1R1_DG_3/T1R3, and T1R1/T1R3_DG_3) were −7.18/−8.05/−8.17/−7.74/−7.14/−7.80/−7.79/−7.56 kcal·mol^−1^. In general, the binding of the short-chain DG _3 to T1R1 and T1R3, respectively, becomes stronger and weaker, while that of the long-chain DG_2 is completely the opposite. In addition, the short-chain EK_4 binds more weakly to T1R1 compared to T1R3, with the opposite being true for long-chain EK_5 (see Figure 11).

It also proves once again that the two pockets of T1R1/T1R3 have a certain preference for the size of the optimal ligands. As for the two modification strategies above, the effect of -OH introduction is slightly better than that of chain length adjustment, which is closely related to the hydrophilic pocket of the T1R1/T1R3 system.

## 3. Materials and Methods

### 3.1. Homology Modeling

Based on sequentially similar template proteins, the primary sequence of a target protein can be converted into a tertiary structure by homology modeling. Commonly utilized homology modeling tools include Modeller, Discover Studio, Spdbvierwer, and some online servers such as SWISS-MODEL and Alpha Fold. In this work, human T1R1/T1R3 amino acid sequences were initially obtained from the UniProt database (Entry: Q7RTX1/Q7RTX0), then the structure of the metabotropic monosodium glutamate receptor (mGluR1, PDB ID: 1EWK) was set as the template protein. Based on the amino acid sequence, the SWISS-MODEL was used for modeling based on the template protein to obtain a successfully constructed human T1R1/T1R3 model. Structural reliability was evaluated using the Discovery Studio 2019 software package and Profile-3D and Ramachandran diagram analyses. The former is mainly employed to assess the compatibility of the primary sequence: the closer the verified score is to the verified expected high score, the higher the quality of the structure being modeled. The latter is mainly used to evaluate the rationality of residue conformations for the modeled structure: the greater the number of residues falling within the conformation allowable region, the more reliable the structure is.

### 3.2. Construction of Dipeptide Structure Database

The successful construction of a dipeptide structure database is a prerequisite for subsequent virtual screening. Through the arrangement and combination of 20 common amino acids, a dipeptide structure database including a total of 400 samples was constructed with the StoneMIND Collector tool and Chem Draw 18.0 software. Relying on optical chemical structure recognition (OCSR) and the International Union of Pure and Applied Chemistry (IUPAC) naming technologies, StoneMIND Collector enables the rapid identification and editing of chemical structures. Molecular structures of 400 dipeptides were optimized using the Chem3D program and MM2 force field, with an energy convergence threshold of 0.0001 kcal/mol. Subsequently, the SwissADME server was utilized to predict physicochemical parameters for all samples in the dipeptide structure database, such as the molecular weight (MW), isoelectric point (PI), net charge at a pH of 7, hydrophobicity value, and so on. As a widely adopted free tool integrating the strategies of BOILED-Egg, iLOGP, and bioavailability radar, the SwissADME server has also been successfully used to predict the pharmacokinetics, drug similarity, and medicinal chemistry friendliness of small molecules, etc. [63]. In addition, the umami prediction probabilities for the database samples are performed by the UMPred-FRL server (http://pmlabstack.pythonanywhere.com/UMPred-FRL, accessed on 20 March 2025). It is an artificial intelligence (AI) meta-model based on the performance training and prediction of 50 methods, which has been widely and successfully applied to the prediction of umami peptide perception. In addition to structural formulae, physicochemical properties, and umami prediction data, the molecular docking results of all dipeptides with T1R1/T1R3 were also added to the database.

### 3.3. Molecular Docking and Virtual Screening

Molecular docking can be utilized to predict complex models of a receptor with a ligand, relying on spatial shape complementarity and energy matching. Based on the Lamarckian genetic algorithm (LGA), AutoDock 4.2 Vina has widely been applied in drug design as a classic molecular docking package. During the conformational search, it considers not only the rotation of small molecules but also the flexibility of protein side-chains. The affinity function was adopted for the docking score, which was provided by fitting the experimental data of the biomacromolecule–organic small molecule system. The smaller the value, the stronger the binding force. The AutoDock Vina package is composed with two key modules—AutoGrid and AutoDock. AutoGrid can preprocess target proteins by assigning charge and position to the grid, while AutoDock is responsible for productive docking between the meshed target protein and its ligand. It is worth mentioning that the grid visualization of affinity in AutoDock Vina favors the subsequent molecular optimization.

In addition to obtaining receptor–ligand complex models, AutoDock Vina is also commonly used for virtual screening, quickly searching large structural databases of small molecule and then mining lead compounds. In this work, AutoDock Vina was initially employed to conduct virtual screening on the dipeptide structure database to mine those with a potential high-umami taste, as well as a good binding ability to T1R1/T1R3. Here, the umami prediction of dipeptides was completed using the UMpred-FRL server. Subsequently, aspartic acid–glycine (i.e., Asp-Gly, DG) and glutamic acid–lysine (i.e., Glu-Lys, EK) dipeptides were screened for subsequent molecular docking. Molecular docking experiments were performed using the AutoDock 4.2 software package, in which the sampling is based on Lamarckian genetic algorithm (LGA), and the scoring adopts the semi-empirical function of the binding free energy [64]. Finally, the screened dipeptides DG and EK were, respectively, docked into the T1R1 and T1R3 pockets of T1R1/T1R3, with the four complex models obtained as T1R1_DG/T1R3, T1R1/T1R3_DG, T1R1_EK/T1R3, and T1R1/T1R3_EK. The size of the docking rectangular box is 40 Å × 40 Å × 40 Å with a grid spacing of 0.375 Å, the box central position of T1R1 was set as X-11.814, Y-10.101, and Z-9.898, and the box central position of T1R1 was set as: X-37.584, Y-(-1.657) and Z-32.895. The parameter of GALS runs was set as 128 in all docking, and the structure with the lowest energy in the largest cluster was taken as the final complex model. After screening, we will check the interactions between ligands and the substrate and T1R1/T1R3 to test the rationality of the molecular docking results.

### 3.4. Molecular Dynamics Simulation

Based on Newton’s second law, statistical mechanics theory, and molecular force fields, molecular dynamics (MD) simulation enables theoretical prediction of the thermodynamic and dynamical properties for biomacromolecules at the atomic level. In this work, five 200 ns comparative MD simulations were carried out for the T1R1_DG/T1R3, T1R1/T1R3_DG, T1R1_EK/T1R3, T1R1/T1R3_EK, and T1R1/T1R3 systems, using the AMBER 20 software package and the ff14SB force field [65]. The solutes were placed in a truncated octahedral box with a boundary of 15.0 angstroms (Å), where the solvent effect was described by the three-point TIP3P water model [66]. A total of 3/3/3/3/3 Na^+^ counter-ions and 29038/29047/29055/29048/29055 water molecules were added to the five systems above, respectively. Additionally, periodic boundary conditions (PBCs) were introduced to reduce the boundary effects of the finite system, achieving the purpose of simulating an infinite system.

Before MD simulations, a two-step energy minimization was carried out to reduce unreasonable geometric collisions in the system. Firstly, a 5000-step steepest descent (SD) and another 5000-step conjugate gradient (CG) minimization were both performed in the solute-constrained state with a constraint force constant of 5 × 10^4^ kcal·mol^−1^·nm^−2^. Subsequently, solute-unconstrained minimization adopting the same two strategies above was also conducted with a convergence criterion of the energy difference between neighboring conformers being less than 1 × 10^−4^ kcal·mol^−1^·nm^−2^. With the completion of energy minimization, a two-step 200 ns comparative MD simulation was then performed: (1) a 5 ns solute-constrained preequilibrium stage where the temperature was gradually increased from 0 to 300 K with a constraint force constant of 1 × 10^−3^ kcal·mol^−1^·nm^−2^; (2) an 195 ns solute-unconstrained productive stage with constant temperature. Throughout the MD simulations, the SHAKE algorithm was used to prevent the destruction of chemical bonds involving non-hydrogen atoms. At the computational level, the truncation radius of non-bond interactions is set to 1 nm and the integration step is 2 fs. Snapshots were sampled every 5000 steps (i.e., 10 ps), and thus, 20,000 conformations were collected for subsequent statistical analyses for each system. Additionally, the conformational motion characteristics of all systems were monitored by the VMD 1.9.3 package [67]. All the molecular dynamics simulation processes were repeated three times, and the results were relatively similar.

### 3.5. Conformational Clustering

Conformational clustering aims to sort out and classify MD simulation trajectories, identifying the representative structure and revealing its significant conformational transition pathways and possible biological function. Conformational clustering not only reduces the redundancy of a large amount of trajectory data, but also saves computing time and provides more intuitive results without losing meaningful structural information. The main idea of conformational clustering is to calculate the root mean square deviation (RMSD) between each conformation, and then build an N × N matrix (N is the number of snapshots). If the RMSD for a specific feature between two arbitrary conformations is less than a preset threshold, they are considered as belonging to the same cluster, where the snapshot with the lowest potential energy is defined as the representative conformation for this cluster. In this work, the RMSD matrix for all C_α_ atomic positions is established, and the formation criterion of conformational cluster is shown in Formula (1).

(1) $$C=\left\{\begin{array}{c} \;1\;\mathrm{if}\;\Delta \mathrm{RMSD}\;\leq \;\mathrm{the}\;\mathrm{threshold}\;\mathrm{value}\\ 0\;\mathrm{if}\;\Delta \mathrm{RMSD}\;>\;\mathrm{the}\;\mathrm{threshold}\;\mathrm{value} \end{array}\right.$$

According to Formula (1), when the ∆RMSD of two conformations is less than or equal to/greater than the threshold value, C is 1/0, respectively, indicating that they belong to the same/different conformational clusters. Here, the calculation formula for RMSD is as follows:

(2) $$RMSD=\sqrt{1/N\sum_{i=1}^{i=N}\delta_{i}^{2}}$$

where N represents the total number of atoms, and δ indicates the offset distance of the corresponding atoms in two conformations.

In this work, conformational clustering was carried out by the MMTSB software package (http://mmtsb.scripps.edu/software/mmtsbToolSet.html, accessed on 28 April 2025) [68], for the 20,000 snapshots, respectively, obtained from the five comparative MD trajectories. In fact, the global and local conformational changes are intuitively presented by superimposing the representative conformations.

### 3.6. Binding Free Energy Calculation

Based on MD trajectories from the T1R1_DG/T1R3, T1R1/T1R3_DG, T1R1_EK/T1R3, and T1R1/T1R3_EK systems, the binding free energies of the receptor with dipeptide ligands were predicted by the solvated interaction energy (SIE) method [69]. Duan mentions that the SIE method can be used to calculate the free energy of drug molecule modification, and the results are good. Therefore, this method has good accuracy and feasibility for drug screening [70]. SIE can provide a more reliable relative binding affinity ranking than the empirical scoring function on a series of different targets and ligands and can more effectively identify active molecules from large compound libraries, especially performing well in the optimization of homologous series compounds, demonstrating its advantages and universality in drug design [71,72]. Based on the 100–200 ns MD trajectories, a total of 100 conformations with the interval of 1 ns were selected to statistically calculate the mean binding free energies via the following Formula 3:

(3) $$∆G_{bind}\left(\rho ,D_{in},\alpha ,\gamma ,C\right)=\alpha \left[E_{c}\left(D_{in}\right)+∆G_{bind}^{R}\left(\rho ,D_{in}\right)+E_{vdw}+\gamma ∆MSA\left(\rho \right)\right]+C$$

where ∆*G*_bind_ (*ρ, D_in_, α, γ, C*) is the binding free energy, which is related to the parameters *ρ, D_in_, α, γ,* and *C.* Specifically, α denotes the global proportionality coefficient characterizing the loss of conformational entropy due to receptor–ligand binding, with a default value of 0.1048. *E_c_* represents the intramolecular Coulomb interactions, and *D_in_* is the internal dielectric constant with a default value of 2.25. $∆G_{bind}^{R}$(*ρ, D*_in_) refers to the change in reaction field energy, which is predicted with the boundary element program BRI BEM. In addition, ρ is the linear proportionality constant of the van der Waals radius with a default value of 1.1; *E*_vdw_ is the intramolecular van der Waals interaction; γ represents the molecular surface area coefficient, which is preset to 0.0129 kcal^−1^·mol^−1^·A^−2^; ∆MSA is the change in molecular surface area; C is the calibration constant, set to −2.89 kcal·mol^−1^.

### 3.7. Weak Interaction Analysis

The interactions within biological macromolecules are mainly categorized into covalent and non-covalent bonds, in which the latter have relatively low potential energy and are also known as weak interactions. These mainly include hydrogen bonding/halogen bonding/π-π stacking/van der Waals forces/steric hindrance, etc. In the theory of atoms in molecules (AIM), the electron density ρ(r) is one of the important parameters evaluating the strength of molecular interactions, which can be expressed in terms of real-space functions. The reduced density gradient (RDG) isosurface was calculated using the MP2/6-311+G** quantum-mechanical (QM) method to characterize the weak interactions [73]. The specific calculation formula is as follows:

(4) $$RDG=\frac{1}{{2\times (3\times \pi^{2})}^{\frac{1}{3}}}\times \frac{\left|\nabla \rho (\gamma )\right|}{{\rho (\gamma )}^{\frac{4}{3}}}$$

where ∇ is the gradient operator and ∇ρ(γ) represents the magnitude of the electron density gradient. Based on the RDG theory and electron density functional, the weak interactions between DG/EK and T1R1/T1R3 pocket residues were compared with the Multiwfn 3.8 software package [74]. All quantum-mechanical calculations, including the wavefunction generation at the MP2/6-311+G** level of theory, were performed using Gaussian 16 (Revision C.01). The resulting wavefunction files were subsequently analyzed using Multiwfn (version 3.8).

## 4. Conclusions

In this work, the structure of the umami peptide receptor T1R1/T1R3 was acquired by homology modeling using the mGluR1 as a structural template, and then its rationality was confirmed by Ramachandran plot and Profile-3D strategies. Sequence alignment and structure superimposition both showed that T1R1/T1R3, like the four major classes of GPCSs, exhibited a low sequence identity and high structural similarity. Next, a natural dipeptide structure database was constructed, and then the representative umami dipeptides DG (Asp-Gly) and EK (Glu-Lys) were screened based on docking scoring and umami prediction. Further, by exploring the molecular recognition of the umami receptors T1R1/T1R3 by DG and EK, respectively, it was found that the two receptor pockets exhibited a certain preference for peptide volume/hydrophilicity/acid–basicity. According to Hole analysis, the T1R1 pocket is much smaller than T1R3, tending to bind the small-volume dipeptide DG, whereas the T1R3 pocket does the opposite. Geometric measurements and pKa calculations both indicate that the T1R3 pocket is generally basic with a larger channel radius, showing a greater preference for the large-volume dipeptide EK.

Based on the comparison of 200 ns MD trajectories between T1R1/T1R3 and its four complex systems, the conformational changes of the receptor and the interaction details with the ligand are both provided. With the binding of the DG and EK dipeptides, the T1R1/T1R3 pocket loops experienced significant conformational closure, which was critical for the subsequent continuous activation of cytoplasmic G proteins. Hydrophilic pocket residues, including A302 and H145′, and channel residues are both involved in a series of conserved hydrogen bonds that contribute to the binding of umami dipeptides. In addition, weak interactions including van der Waals forces from L75 and T167′, as well as steric hindrance dominated by S172, all assisted in the tight binding of umami-peptides to T1R1/T1R3 pockets. In the last section of this article, DG and EK dipeptides were, respectively, modified by introducing an -OH group and adjusting the chain length with eight candidate molecules being obtained, and finally the design effectiveness was evaluated via the calculation of binding free energies. This work not only reveals the molecular recognition and conformational change of the T1R1/T1R3 receptor with the umami dipeptides DG/EK, showing a certain theoretical significance, but also provides molecular design suggestion of umami dipeptides, having definite application value.

## Data Availability

All data generated or analyzed during this study are included in this published article (and its Supplementary Materials).

## Supplementary Materials

The following supporting information can be downloaded at https://www.mdpi.com/article/10.3390/molecules30132774/s1, Figure S1: Sequence alignment of T1R1/T1R3 and mGluR1 crystal structure (PDB ID: 1EWK); Figure S2: Geometric data of the length, area and volume for T1R1(A)/T1R3(B) pockets and DG/EK(C); Figure S3: Convergence analysis of MD trajectories for the T1R1/T1R3, T1R1_DG/T1R3, T1R1/T1R3_DG, T1R1_EK/T1R3 and T1R1/T1R3_EK systems; Figure S4: The interactions between DG/EK and T1R1/T1R3 pockets at three different simulation times (i.e., 10, 100 and 200 ns) for the T1R1_DG/T1R3 (A-C), T1R1/T1R3_DG (D-F), T1R1_EK/T1R3(G-I) and T1R1/T1R3_EK(J-L) systems; Table S1: Physicochemical parameters, binding energies, and umami prediction probabilities for 33 experimentally reported umami dipeptidesa; Table S2: Contact residues on all channels of T1R1/T1R3; Table S3: The pKa values of titrable residues on all channels of T1R1/T1R3; Table S4: The stable hydrogen bonds between residues around DG/EK and T1R1/T1R3 pocket are arranged in descending order of frequency.
