# Supplementary material for: Molecular Recognition and Modification Strategies of Umami Dipeptides with T1R1/T1R3 Receptors
Source: Molecules. 2025 Jun 27;30(13):2774. doi: 10.3390/molecules30132774 (PMC12251166; doi:10.3390/molecules30132774)
Supplement: Supplementary file 1 [file molecules-30-02774-s001.zip › molecules-3715008-supplementary.pdf]

Molecular recognition and modification strategies of umami dipeptide with T1R1/ T1R3 receptors

Kaixuan Hu[1,2 †], Guangzhou Sun[2, †], Wentong Yu[2], Mengyu Zhang[2], Shuang Wang[2], Yujie Cao[2], Dongling Hu[2], Li Liang[2], Gang He[2,*], Jianping Hu[2,*] Wei Liu[1,*]

[1] Bamboo & Forest Institute of Science, Technology and Industrial Innovation, Leshan Normal University, Leshan 614004, China

[2] Key Laboratory of Medicinal and Edible Plants Resources Development of Sichuan Education Department, School of Pharmacy, Chengdu University, Chengdu 610106, China;

[†] These authors contributed equally to this work.

[*] Correspondence: hegang@cdu.edu.cn (Gang He); hjpcdu@163.com (Jianping Hu); liuweicdu@sina.com (W.L.)

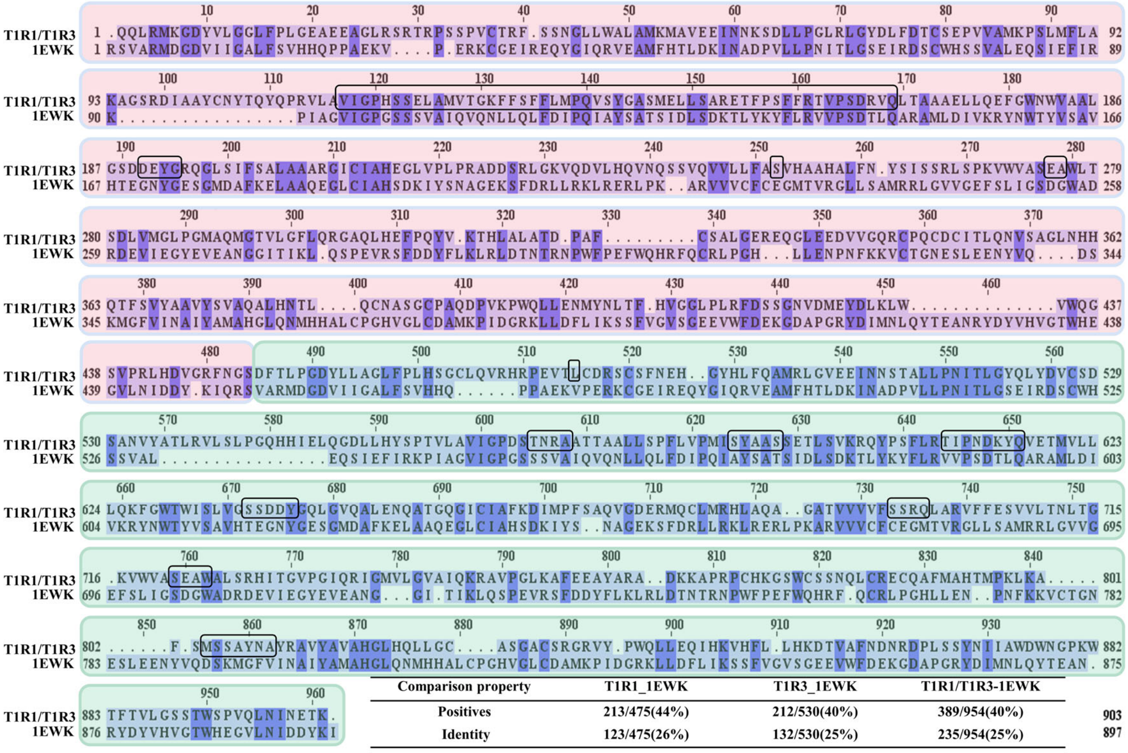

Figure S1. Sequence alignment of T1R1/T1R3 and mGluR1 crystal structure (PDB ID: 1EWK). Pink and green are respectively used to represent the sequence alignment of T1R3 and T1R3 with 1EWK; blue represents the same amino acids of T1R1/T1R3 and 1EWK; black boxes are used to represent the pocket residues involved in substrate recognition in T1R1/T1R3.

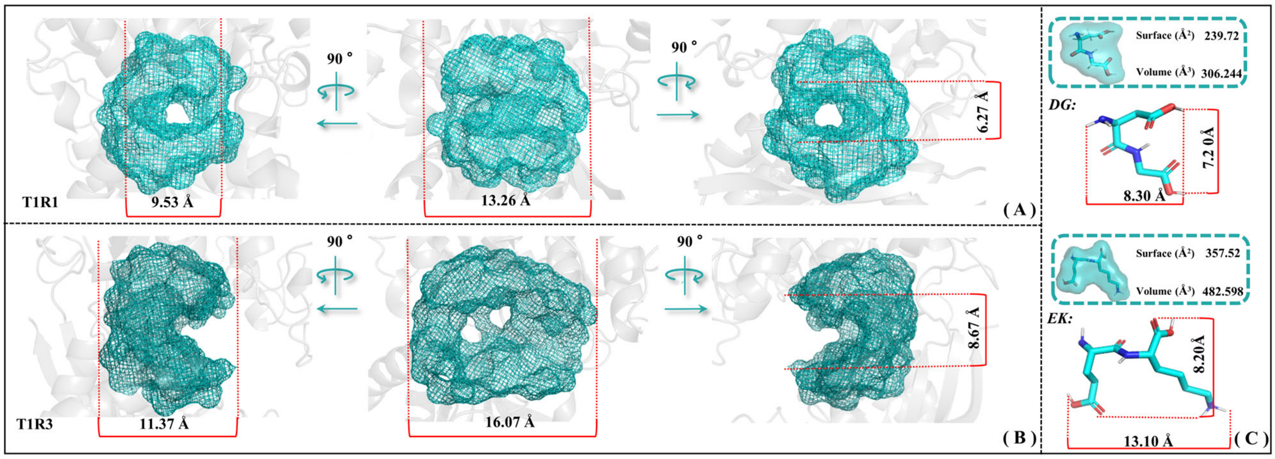

Figure S2. Geometric data of the length, area and volume for T1R1(A)/T1R3(B) pockets and DG/EK(C). On the whole, the sizes of T1R1 pocket and EK are significantly larger than those of T1R3 and EK, respectively.

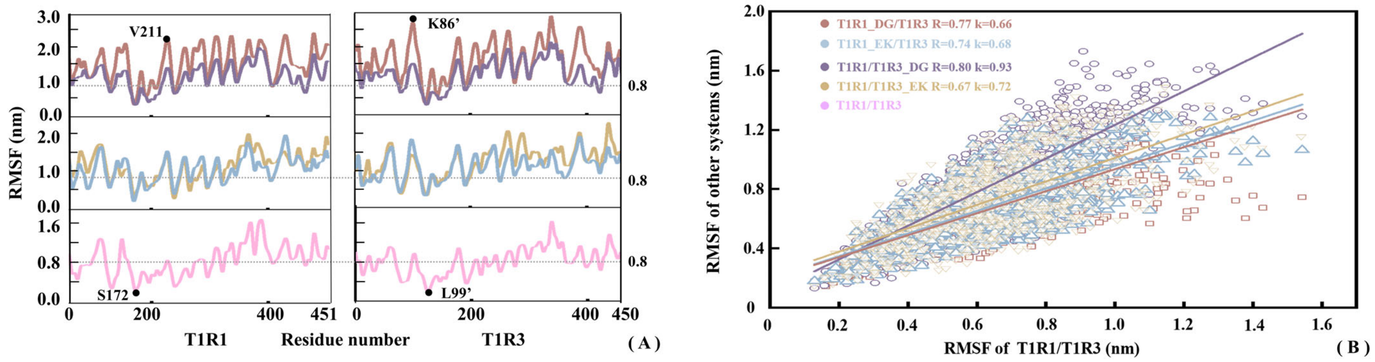

Figure S3. Convergence analysis of MD trajectories for the T1R1/T1R3, T1R1_DG/T1R3, T1R1/T1R3_DG, T1R1_EK/T1R3 and T1R1/T1R3_EK systems. (A) RMSF distribution at the residue level; (B) Comparison of RMSF correlation between T1R1/T1R3 and the other four complex systems.

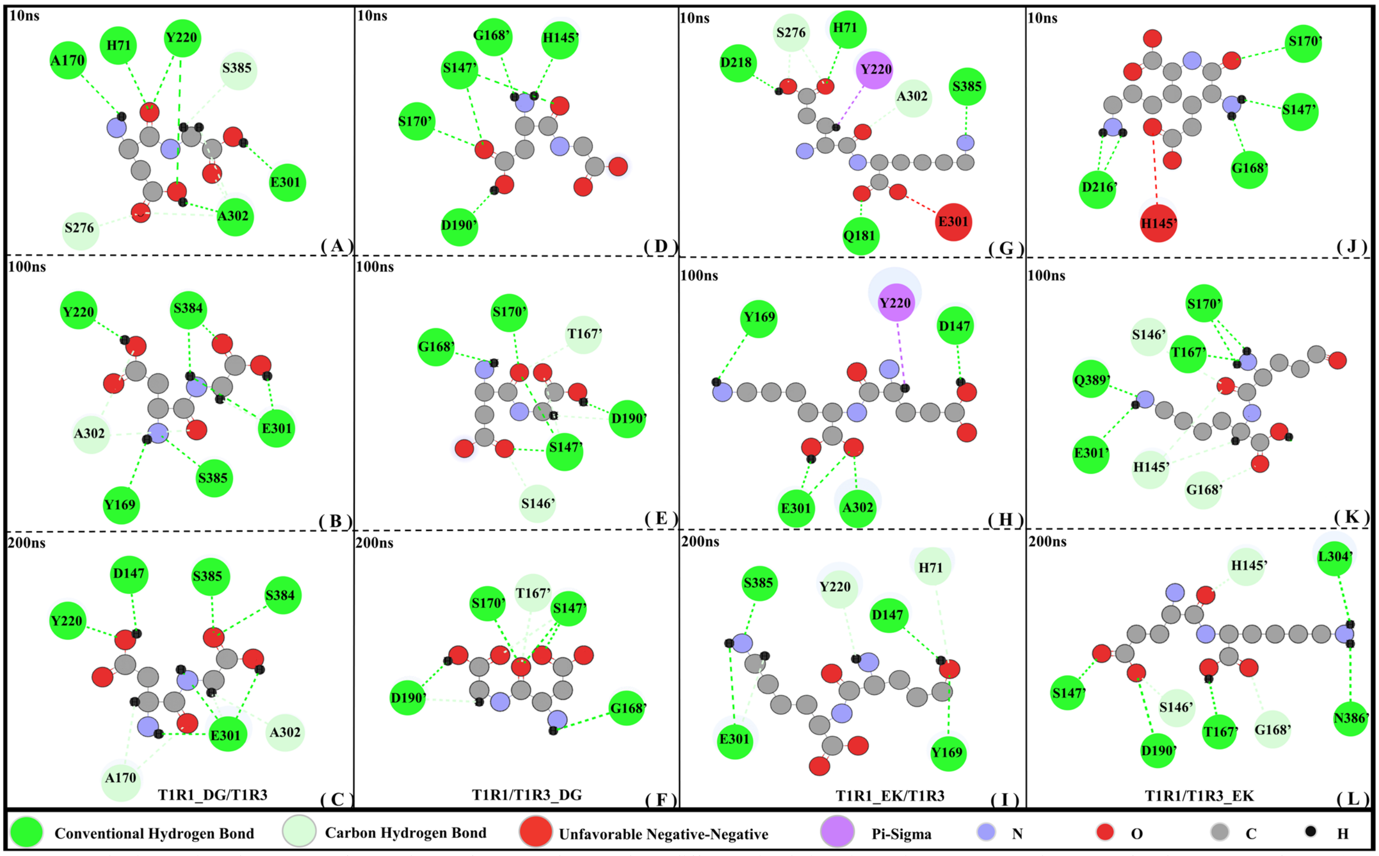

Figure S4. The interactions between DG/EK and T1R1/T1R3 pockets at three different simulation times (i.e., 10, 100 and 200 ns) for the T1R1_DG/T1R3 (A-C), T1R1/T1R3_DG (D-F), T1R1_EK/T1R3(G-I) and T1R1/T1R3_EK(J-L) systems.

Table S1. Physicochemical parameters, binding energies, and umami prediction probabilities for 33 experimentally reported umami dipeptides[a]

Dipeptides	[b] TIR1_*/T1R3	[c] T1R1/T1R3_*	[d] PI	[e] MW	Net charge	Hydrophobicity	[f] Probability	[g] Volume
EG	-6.8(-6.5)	-5.7(-5.3)	3.85	204.18	-1.02	-1.95	0.984	408.597
[h] ***EK***	***-7.1(-7.3)***	***-8.3(-8.1)***	***6.41***	***275.3***	***-0.02***	***-3.7***	***0.984***	***549.544***
AE	-6.8(-6.9)	-5.8(-6.0)	3.75	218.21	-1.02	-0.85	0.983	432.977
VE	-6.8(-6.6)	-7.5(-7.4)	3.85	246.26	-1.02	-0.35	0.982	482.867
EN	-7.1(-6.9)	-5.7(-5.7)	3.85	261.23	-1.02	-3.50	0.980	483.870
AD	-5.7(-6)	-5.9(-5.8)	3.75	204.18	-1.02	-0.85	0.978	401.751
GD	-6.1(-6.3)	-6.5(-6.6)	3.75	190.15	-1.02	-1.95	0.976	376.565
VD	-6.9(-7)	-7(-7.2)	3.75	232.23	-1.02	0.35	0.972	451.491
DA	-6.6(-6.7)	-6.7(-6.6)	3.75	204.18	-1.02	-0.85	0.971	399.968
DK	-6.9(-6.9)	-6.1(-6)	6.34	234.21	-0.02	-3.70	0.969	516.135
EV	-6.7(-6.9)	-5.7(-5.6)	3.85	246.26	-1.02	-0.35	0.966	481.822
[h] ***DG***	***-8.1(-7.9)***	***-7.3(-7.6)***	***3.75***	***190.15***	***-1.02***	***-1.95***	***0.962***	***375.220***
GE	-6.5(-6.6)	-5.8(-6)	3.85	204.18	-1.02	-1.95	0.961	408.917
TE	-6.8(-6.7)	-7(-6.8)	3.85	248.23	-1.02	-2.10	0.958	470.934
ED	-6.9(-7)	-7(-7.1)	3.56	262.22	-2.02	-3.50	0.953	479.064
SD	-6.3(-6.1)	-6(-6.2)	3.75	220.18	-1.02	-2.15	0.951	415.038
EL	-6.8(-6.8)	-5.9(-6)	3.85	260.29	-1.02	-0.15	0.948	510.555
ES	-6.7(-6.6)	-6.2(-6.1)	3.85	234.21	-1.02	-2.15	0.948	445.166
VG	-6.6(-6.4)	-6.5(-6.8)	6.11	174.2	-0.02	1.90	0.946	381.778
AH	-7.1(-7)	-6.9(-6.6)	7.56	226.23	0.21	-0.70	0.893	448.303
EP	-7.3(-7)	-6.2(-6.2)	3.85	244.24	-1.02	-2.55	0.891	464.416
DE	-6.8(-6.9)	-7.1(7.2)	3.56	262.22	-2.02	-3.50	0.887	476.471
DD	-6.9(-6.6)	-6.1(6.2)	3.5	248.19	-2.02	-3.50	0.848	445.561
EE	-7(-6.7)	-6(-6.4)	3.62	276.24	-2.02	-3.50	0.759	510.114
LV	-6.7(-6.8)	-6(-6.1)	6.11	230.3	-0.02	4.00	0.731	487.165
HV	-7.5(-7.3)	-6.2(-6)	7.56	254.29	0.21	-0.50	0.689	500.170
LE	-7.2(-7)	-7.7(-7.4)	3.85	260.29	-1.02	-0.15	0.530	511.650
DL	-6.9(-6.6)	-5.9(-6.3)	3.75	246.26	-1.02	-0.15	0.510	477.627
NY	-8.2(-8.1)	-7.1(-7)	9.36	295.29	0.97	-2.20	0.412	564.688
ID	-7(-7.2)	-6(-6.2)	3.75	246.26	-1.02	-0.50	0.408	479.733
RL	-7.5(-7.2)	-6.1(-5.9)	10.56	287.36	0.98	-0.35	0.230	586.337
IE	-7(-7.1)	-7.7(-7.4)	3.85	260.29	-1.02	-0.50	0.185	515.950
QL	-7.1(-7.2)	-7.3(-7.1)	6.11	259.3	-0.02	-0.15	0.150	518.365

[a] The dipeptides are listed in descending order of predicted umami probability. Docking energies of umami dipeptides respectively to [b] T1R1 and [c] T1R3 pockets with unit of kcal·mol^{-1}(The figures in parentheses represent the docking energies obtained from GOLD); [d] Isoelectric point; [e] Molecular weight with unit of Da; [f] Umami prediction probability; [g] Molecular volume with unit of nm^3; [h] Bold italics are used to indicate representative umami dipeptides screened for the subsequent investigation of molecular recognition; * DG or EK.

Table S2. Contact residues on all channels of T1R1/T1R3.

Systems	Contact residues
T1R1	S384, I326, A325, *L305*, *S385*, A304, *E301*, H71, A302, *D147*, A170, *Y220*, S148, S276, F247, D218, S107, R151, D219, N150, *L75*, S217, Q222, Q181, E70, Q226, *E301*, T233, H308, R180, A330, F381, *S306*, V465, K335, K460, K482, P332, K434, M371, K343, *Y169*, D192
T1R3	S104’, A46’, E47’, L245’, D216’, R252’, R54’, D215’, E105’, P244’, L255’, *D190’*;<br>^{I}L468’, ^{I}G328’, ^{I}Q379’, ^{I}L325’, ^{I}*N386’*, ^{I}T305’, ^{I}H388’, ^{I}Y394’, ^{I}E301’, ^{I}L304’, ^{I}*Q389’*, ^{I}*S170’*, ^{I}*E148’*, ^{I}H145’, ^{I}A302’, ^{I}*S146’*, ^{I}Y218’, ^{I}E217’, ^{I}R220’, ^{I}E240’, ^{I}S224’, ^{I}A329’, ^{I}Y454’, ^{I}R185’, ^{I}A383’, ^{I}H332’, ^{I}D470’, ^{I}G439’, ^{I}L440’, ^{I}*S147’*;<br>^{II}N68’, ^{II}E45’, ^{II}H278’, ^{II}Q359’, ^{II}R357’, ^{II}D307’, ^{II}L308’, ^{II}R247’, ^{II}A248’, ^{II}D249’, ^{II}*T167’*, ^{II}*G168’*, ^{II}Q368’, ^{II}L312’, ^{II}G367’, ^{II}V366’, ^{II}V365’, ^{II}R52’, ^{II}T55’

T1R1 and T1R3 contain 1 and 2 channels respectively, with 43 and 42/31 contact residues. For T1R3, the superscript I in the upper left corner indicates the residues unique to Channel I; the superscript II in the upper left corner shows the residues unique to Channel II; those without any identification indicate the common residues for Channels I and II. In addition, italics is used to indicate the residues located in the substrate binding pocket of T1R1 or T1R3.

Table S3. The pKa values of titrable residues on all channels of T1R1/T1R3.

**Systems**	**Residues/ pKa**	**Sum**	**Average**
T1R1 Channel	D147/4.41, D192/6.73, D218/5.37, D219/4.83, E70/6.39, E301/8.78, H71/1.19, H308/3.94, Y169/14.41, Y220/ 15.21, R151/11.32	82.58	7.51
T1R3 Channel I	D190’/5.25, D215’/2.91, D216’/4.00, E47’/3.96, E105’/4.84, Y394’/12.13, R252’/13.52, R54’/13.62; ^{I}R220’/15.04, ^{I}E148’/4.46, ^{I}E217’/3.20, ^{I}E301’/7.50, ^{I}Y218’/15.25, ^{I}R185’/10.20, ^{I}Y454’/15.18	131.06	8.74
T1R3 Channel II	D190’/5.25, D215’/2.91, D216’/4.00, E47’/3.96, E105’/4.84, Y394’/12.13, R252’/13.52, R54’/13.62; ^{II}D307’/3.54, ^{II}E45’/4.92, ^{II}R247’/13.22, ^{II}T167’/15.13, ^{II}R357’/14.38	111.42	8.57

T1R1 and T1R3 are composed of 1 and 2 molecular channels respectively, containing 11/15/13 titratable residues. For T1R3, the superscript I in the upper left corner indicates the residues unique to Channel I; the superscript II in the upper left corner shows the residues unique to Channel II; those without any identification indicate the common residues for Channels I and II.

Table S4. The stable hydrogen bonds between residues around DG/EK and T1R1/T1R3 pocket are arranged in descending order of frequency.

Systems	Acceptors	Donors	Frequency (%)	Distance (nm)	Angle （°）
**T1R1_DG/ T1R3**	MOL904@O1	***S385@OG_HG***	92.17	2.7216	163.4482
	L43@O	***Y169@OH_HH***	89.04	2.7515	159.3171
	D218@O	***Y220@N_H***	83.64	2.8267	149.2009
	E301@O	MOL904N1_@H6	79.71	2.8500	156.949
	A170@O	D192@N_H	79.24	2.8489	159.1094
	A302@OD2	A170@OG_HG	74.15	2.666	165.1167
	D192@OD2	A170@OG_HG	74.15	2.666	165.1167
	H71@O	***L75@N_H***	73.64	2.8618	157.3185
	MOL904@O3	***S306@OG_HG***	72.84	2.7635	160.9001
**T1R1/ T1R3_DG**	***D190'@OD2***	MOL904@O2_H9	98.07	2.5938	164.4807
	MOL904@O1	***S147'@OG_HG***	75.57	2.7032	156.0289
	H145'@O	***E148'@N_H***	75.12	2.6318	156.4529
	MOL904@O4	***G168'@N_H***	51.08	2.8654	162.5111
	D216'@O	G219'@N_H	46.95	2.8856	155.2955
**T1R1_EK/ T1R31**	A302@O	R277@NH2_HH21	89.21	2.8108	158.2306
	S385@O	***N388@N_H***	80.82	2.8371	155.0786
	E301@O	MOL904@N1_H6	79.71	2.85	156.949
	MOL904@N2	***S385@OG_HG***	79.62	2.7606	165.3895
	S385@OG	I326@N_H	65.73	2.8843	161.9177
	H71@ND1	G72@N_H	52.45	2.9065	157.9764
	D147@OD2	MOL904@O_H	50.35	2.6476	155.7283
**T1R1/ T1R1_EK**	***D190'@OD2***	***S170'@OG_HG***	97.41	2.6529	165.7709
	S147'@OD1	R185'@NH2_HH21	94.01	2.7713	155.2609
	G168'@O	R185'@NH1_HH11	93.35	2.7819	153.878
	L40'@O	***T167'@OH_HH***	84.23	2.782	158.0941
	T167'@O	MOL904@O3_H10	80.24	2.7601	161.0336
	H145'@O	***G168'@N_H***	78.24	2.8518	157.3209
	S170'@O	***D190'@N_H***	76.25	2.8732	163.6028
	S146'@O	MOL904@N_H6	66.67	2.8712	161.9869
	H145'@O	MOL904@N_H6	66.67	2.8712	161.9869
	MOL904@N_H6	***Q389'@N_H***	65.27	2.8685	149.5669

Italic and bold both are used to represent T1R1/T1R3 pocket residues (see Figure 4). In fact, a large number of channel residues are also found to form hydrogen bonds with DG/EK. (see Table S2).
